# Prevalence and Correlates of Significant Posttraumatic Stress Disorder Symptoms in Children in Ukraine

**DOI:** 10.1016/j.jaacop.2026.04.007

**Published:** 2026-05-01

**Authors:** Sanja Lestarevic Stojkov, Marija Lero, Svitlana Drin, Rachel Calam, Andrea Danese, Aala El-Khani, Martha Fetsyk, Halyna Nesmashna, Halyna Radchuk, Anna Tarasenko, Kateryna Yavna, John R. Weisz, Dennis Ougrin

**Affiliations:** a Institute of Mental Health, Belgrade, Serbia; b School of Business, Örebro University, Örebro, Sweden; c National University of Kyiv-Mohyla Academy, Kyiv, Ukraine; d University of Manchester, Manchester, United Kingdom; e Social, Genetic and Developmental Psychiatry Centre, Institute of Psychiatry, Psychology and Neuroscience, King’s College London, London, United Kingdom; f Institute of Psychiatry, Psychology and Neuroscience, King’s College London, London, United Kingdom; g National and Specialist CAMHS Clinic for Trauma, Anxiety, and Depression, South London and Maudsley NHS Foundation Trust, London, United Kingdom; h Prevention, Treatment and Rehabilitation Section, United Nations Office on Drugs and Crime (UNODC), Vienna, Austria; i University of Manchester, Manchester, United Kingdom; j University of Bath, Bath, United Kingdom; k Ternopil Communal Methodological Centre for Scientific-Educational Innovations and Monitoring, Ternopil, Ukraine; l Ternopil Volodymyr Hnatyuk National Pedagogical University, Ternopil, Ukraine; m Queen Mary University, London, United Kingdom; n Ukrainian Institute of CBT, Lviv, Western Ukrai; o Children and War Foundation, London, United Kingdom; p Harvard University, and Harvard Medical School, Cambridge, Massachusetts; q Youth Resilience Unit, Wolfson Institute of Population Health, Queen Mary University of London, United Kingdom; r Institute of Mental Health, Ukrainian Catholic University, Ukraine

**Keywords:** war trauma, posttraumatic stress disorder, children, adolescents, screening

## Abstract

**Objective:**

Childhood traumatic events are key risk factors for posttraumatic stress disorder (PTSD) and other psychiatric conditions. The war context of the Russian invasion of Ukraine has exposed Ukrainian children to direct and indirect war-related traumatic events and has significantly increased the mental health burden of Ukrainian youth. The objective of the present article is to report the prevalence and correlates of significant PTSD symptoms among children in a part of Ukraine that is not directly affected by the war.

**Method:**

This is a cross-sectional study conducted in Ternopil. All primary schools in Ternopil were contacted, and children and adolescents aged 7 to 17 years were included in the study if the legal guardian did not object to participation. The Child Revised Impact of Events Scale (CRIES-8) was completed by the youth, whereas demographic characteristics were collected from the youth, parents/legal guardians, and teachers.

**Results:**

A total of 4,753 children and adolescents from 26 schools, aged 7 to 17 years (mean age 11.03 ±1.70 years), 52.3% female, participated in this study. The prevalence of clinically significant PTSD symptoms, defined as CRIES-8 scores of 17 or above, was 40.3% in the overall sample. Female participants had significantly higher intrusion, avoidance, and total CRIES-8 scores (*p* < 0.001) compared to male participants. The highest CRIES-8 scores were observed in the youngest age group (7-10 years).

**Conclusion:**

The study shows a high prevalence of significant PTSD symptoms even among Ukrainian children and adolescents living in areas far from active conflict zones, suggesting an urgent need for solutions to protect and improve the mental health of youth in war-affected countries.

**Clinical guidance:**

• Clinicians should consider screening children from war-affected countries for trauma-related symptoms, even if they live far from active conflict zones, as significant stress may be present without direct exposure.

• Younger children may require special attention, as early school-age youth appear to be particularly vulnerable to war-related stress and may exhibit PTSD symptoms differently than adolescents.

• Girls may require more targeted monitoring and early intervention, given the consistently higher levels of trauma-related symptoms in this group.

**Study registration information:**

Evaluation of the Teaching Recovery Techniques plus Parenting, a Cluster Randomised Controlled Trial in Ukrainian Schools in Ternopil (TRUST); https://doi.org/10.1186/ISRCTN15476405.

**Diversity & Inclusion Statement:**

We worked to ensure sex and gender balance in the recruitment of human participants. We worked to ensure race, ethnic, and/or other types of diversity in the recruitment of human participants. We worked to ensure that the study questionnaires were prepared in an inclusive way. Diverse cell lines and/or genomic datasets were not available. While citing references scientifically relevant for this work, we also actively worked to promote sex and gender balance in our reference list. While citing references scientifically relevant for this work, we also actively worked to promote inclusion of historically underrepresented racial and/or ethnic groups in science in our reference list.

The author list of this paper includes contributors from the location and/or community where the research was conducted who participated in the data collection, design, analysis, and/or interpretation of the work.

## Plain language summary

The authors studied the mental health of over 4,700 school children in Ternopil, a region of Ukraine far from active fighting. Using a short questionnaire on war-related stress, the authors found that around 40% of them had symptoms that indicated they were at risk of posttraumatic stress disorder (PTSD), especially younger children and girls. The results suggest that war may impact the mental health of children even in relatively safer areas and that widespread accessible psychological support may be urgently needed.

Childhood traumatic events are key risk factors for psychiatric disorders,^1^^,^^2^ potentially leading to the development of posttraumatic stress disorder (PTSD).^1^^,^^2^ Traumatic events disrupt an individual’s usual sense of control over life, going beyond what is considered a normal human experience, and are frightening, dangerous, or violent, representing a threat to life or bodily integrity.^3^ Moreover, witnessing events that threaten the life or physical safety of loved ones or even learning about traumatic experiences involving loved ones can also be traumatic,^3^ and this can be especially important for children because their well-being depends on their perceived security in their environment.^4^

A diagnosis of PTSD significantly increases the likelihood of developing comorbid psychiatric conditions, leading to complex psychopathology such as depressive disorder, self-harm, suicide attempts, or externalizing disorders such as conduct problems and substance abuse.^1^ This highlights the strong connection between PTSD risk and the factors present in the youth recovery environment, and underscores the importance of developing effective posttraumatic screening tools and interventions for children and adolescents.^2^

The 21st century has brought several conflicts that have significantly affected the civilian population and increased the risk of experiencing consequences that are harmful to mental health.^5^ The Russian invasion of Ukraine has caused social, political, and financial instability worldwide, leading to a decline in global well-being.^6^ It is estimated that around 7.5 million children are at extreme risk for mental and physical health issues because many of their essential needs cannot be met, including physical safety, food, shelter, education, and social support.^7^ Two years after the escalation of conflict in Ukraine in 2014, a school-based survey found that all adolescents in both war-affected Donetsk and non–war-affected Kirovohrad regions had experienced at least one war-related event.^5^ In Donetsk, around 60% witnessed armed attacks, 30% saw civilians harmed or intimidated, 28% were displaced, and 14% experienced direct victimization. In Kirovohrad (now called Kropyvnytskyi), direct exposure was rare (<1%), but many adolescents still reported non-violent disruptions such as loss of social connections or adaptation difficulties.^5^ Even in this non–war-affected region, adolescents showed elevated rates of anxiety and depression, likely reflecting fear, concerns for the safety of others, and continued media exposure to the ongoing conflict.^5^ Upon the escalation of the conflict in 2022, there has been a notable increase in parent-reported uneasiness and worry about their children.^8^ The mental health burden of Ukrainian youth is substantial, as the prevalence of clinically significant PTSD symptoms among adolescents is estimated at 35%.^9^

The objective of the present paper is to report the prevalence of PTSD symptoms among children in a part of Ukraine that is not directly affected by the war, but where witnessing war-related traumatic events or learning about them could have had a detrimental impact on the risk for symptoms of PTSD.

## Method

### Study Design and Participants

This cross-sectional analysis is part of an ongoing intervention study that we prospectively registered (https://www.isrctn.com/ISRCTN15476405). The intervention study aims to evaluate whether the community-based intervention called Teaching Recovery Techniques Plus Parenting (TRT+P) program influences child mental health, specifically symptoms of posttraumatic stress, in comparison to the mental health in children with similar demographic characteristics who receive only services as usual. For the present study, we contacted all primary schools in Ternopil, a city in western Ukraine that has not been directly affected by war. Schools were randomly allocated to one of the 2 possible groups: (1) the intervention group offering the TRT+P program, and (2) the waitlist control group receiving services as usual, followed by the TRT+P program around 20 weeks later. Outcome data will be collected at 3 points: pre-intervention (T1), post-intervention (T2; about 8 weeks after randomization), and at follow-up (T3; about 20 weeks after randomisation). The study will primarily measure changes in self-reported child mental health, specifically symptoms of PTSD. This prevalence study is derived from the data obtained at pre-intervention point of the outcome assessment.

Schools were chosen because school screening is a promising tool for detecting adolescents at risk for mental health problems and providing further implementation of help. All parents or legal guardians were informed about the research; participation followed an opt-out consent procedure, whereby children and adolescents aged 7 to 17 years were included unless a legal guardian objected and a parent or other primary caregiver wanted to participate. Youth at high risk for harm to self or others were excluded based on the assessment of school psychologists, with safeguarding referrals made as appropriate.

At the time of the study, Ternopil was located at a considerable distance from active conflict zones in Ukraine. Children in the sample were therefore rarely directly exposed to bombardment or immediate physical danger, although many experienced indirect effects such as disrupted routines caused by frequent air raid sirens necessitating hiding in air raid shelters, media exposure to the conflict, and stress within their families and the community, especially related to deaths on the front line. A minority of participants had arrived from front line areas or regions with higher risk, and their experiences are noted where relevant. For clarity, the term “not directly exposed” is used throughout the manuscript to describe children and adolescents who were living outside of immediate danger zones but were still affected by the broader consequences of the conflict.

Data were collected using a secure online platform, Google Forms, from December 2022 to November 2024. The study was approved by the Ethics Committee of Ternopil Volodymyr Hnatiuk National Pedagogical University (approval ID01112, granted November 20, 2023).

### Measures

To measure symptoms of PTSD, the self-report instrument, the 8-item Children’s Revised Impact of Events Scale (CRIES-8),^10^ was completed by the children and adolescents participating in the study. The CRIES-8 is designed as a screening tool to identify children and adolescents at risk for PTSD^11^ so as to offer them suitable and timely treatment. The self-reported CRIES-8 is a brief measure, quick to complete and user-friendly for children.^11^ It explores the presence of PTSD symptoms in the past 7 days. It is free and can be downloaded from the Children and War Foundation`s website.^12^ The instrument consists of 4 items measuring intrusion and 4 items measuring avoidance, with scoring ranging from 0 to 5 depending on the expressed symptoms (0 = not at all, 1 = rarely, 3 = sometimes, 5 = often). The instrument has not been validated in Ukrainian but has been widely used in previous research. The psychometric properties of the CRIES-8 have demonstrated it to be a reliable and valid measure with a stable factor structure.^13^ Factor analytic studies consistently confirm a stable 2-factor structure, with items loading on the intrusion and avoidance subscales, demonstrating good internal consistency and allowing the measure to be used in both total and subscale form. The CRIES-8 is as effective for screening as the CRIES-13, differing only in the absence of arousal items.^10^ A cutoff score of 17 provides the best balance between sensitivity and specificity, correctly classifying 78% to 81% of all children according to a study^14^ conducted in a clinical population of adolescents in Denmark.

Demographic characteristics were collected from children and adolescents, parents or legal guardians, and teachers. The questions included the child’s or adolescent’s age, number of children or adolescents in the family, the child`s or adolescent’s academic performance (low, intermediate, high), who takes care of the child or adolescent (single parent or not), whether parents are in the military and if so, which parent (mother or father), whether there have been changes in residence (yes vs no), and whether the family has relocated because of war (yes vs no). In addition, the age of the informant or caregiver, the marital status of the informant or caregiver (married or not, widow vs widower, or other), the informant or caregiver’s education (primary, secondary, tertiary), employment status (permanent employment, yes vs no), ethnicity (Ukrainian vs other), religion (Christianity vs other religion vs other) or spoken language (Ukrainian vs Russian vs both vs other) of the informant or caregiver were also collected.

### Statistical Analysis

We used descriptive statistics to summarize demographic variables (age, sex, ethnicity, number of children or adolescents in the family, academic performance, growing up in dual or single parent families, parents’ military involvement, displacement, education, employment, and marital status of the informant or caregiver) and the clinical outcome measured by the CRIES-8 (intrusion, avoidance, and total scores). CRIES-8 reliability was examined using the Cronbach α. For each sociodemographic variable (sex, grade, number of children or adolescents in the family, age, and age group), the Levene test was used to assess homogeneity of variance. Where variances or group sizes were unequal, Welch *t* tests (for 2 groups) and the Welch analysis of variance (ANOVA) (for more than 2 groups) were applied. In addition, Kruskal–Wallis tests were performed to confirm the nonparametric results. The Pearson correlation coefficient was calculated for associations with continuous age. Age was analyzed in 3 revised categories (age 7-10, 11-13, and 14-17 years) to reduce heteroscedasticity observed when age was treated as continuous. This grouping stabilized variance and allowed ANOVA assumptions to be met, although results are interpreted descriptively. All statistical analyses were performed using SPSS.

## Results

### Participant Characteristics

A total of 27 schools were contacted, with the total student population of 15,214 children. Official refusal to participation was given by the caregivers of 12 children, whereas the number of children who failed to complete the survey without explicit refusal was 10,449.

A total of 4,753 children from these 27 schools (31.24% of the total student population in the contacted schools), aged 7-17 years (mean age = 11.03 ± 1.70 years), 52.3% female, participated in this study. Demographic information about the participants was gathered from 1,512 parents and 1,815 teachers. The sociodemographic characteristics of the overall sample are summarized in Table 1.

**Table 1:** Sociodemographic Characteristics of the Overall Sample

	n	%
Sex assigned at birth		
Male	2,265	47.7
Female	2,488	52.3
Age of child, y		
7-10	1,820	38.3
11-13	2,625	55.2
14-17	308	6.5
Informant/caregiver’s relation to child		
Mother	1,449	95.8
Father	56	3.7
Grandmother	3	0.2
Guardian	2	0.1
Sister	1	0.1
Marital status of informant/caregiver		
Married	1,310	86.8
Not married	143	9.5
Divorced	40	2.6
Widowed	16	1.1
Civil marriage	3	.2
Child cared for by single parent		
Yes	141	7.8
No	1,674	92.9
Child’s father is in the army		
Yes	151	8.3
No	1,664	91.7
Child’s mother is in the army		
Yes	1	0.1
No	1,814	99.9
Informant/caregiver’s education		
Primary	5	0.3
Secondary	361	23.9
High	1,146	75.8
Permanent employment of informant/caregiver		
Yes	1,118	73.9
No	394	26.1
Change of family residence		
Yes	304	20.1
No	1,208	79.9
Family displaced because of war (DBW)		
Yes	86	4.7
No	1,729	95.3
Ethnicity of informant/caregiver		
Ukrainian	1,508	99.7
Other	4	0.3
Religion of informant/caregiver		
Christianity	1,477	97.7
Non-religious	25	1.7
Other religion	10	0.7
Spoken language of informant/caregiver		
Ukrainian	1,459	96.5
Both Ukrainian and Russian	49	3.2
Russian	4	0.3
No. of children in family		
1	381	25.3
2	860	57.1
3	219	14.5
4	34	2.3
5	6	0.4
>5	7	0.4
Academic performance of child		
High	673	37.1
Intermediate	1,055	58.1
Low	87	4.8

### Reliability of CRIES

The internal consistency of CRIES-8 was found to be within the good range in this sample (Cronbach α = .825). The item-total correlations for all items are presented in Table S1, available online.

### Prevalence of Clinically Significant PTSD Symptoms

A CRIES-8 score of 17 or above is considered to be a positive screening for PTSD. In our sample, the prevalence of significant PTSD symptoms was estimated to be 40.3% in the overall sample. The prevalence of significant PTSD symptoms in different sex and age groups is indicated in Table 2.

**Table 2:** Prevalence of Significant Posttraumatic Stress Disorder (PTSD) Symptoms in Sample

	n	%
Overall sample	1,915	40.3
Male	815	36.0
Female	1,100	44.2
Age 7-10 y	838	46.0
Age 11-13 y	969	36.9
Age 14-17 y	108	35.1

### Analysis of CRIES-8 Scores by Sociodemographic Variables

The mean CRIES-8 total scores in different sex, age, and grade subgroups are indicated in Table 3. The CRIES-8 intrusion and avoidance subscores and their differences in different sociodemographic subgroups are indicated in Table S2, available online.

**Table 3:** Child Revised Impact of Events Scale (CRIES-8) Total Scores and Their Differences in Sociodemographic Subgroups

	CRIES-8 total		
	Mean ± SD	*p*	
Sex assigned at birth			
Male	13.20 ± 9.67	.000	
Female	14.91 ± 9.86		

	CRIES-8 total		
	Mean ± SD	*F*	*p*
Age group, y			
7-10	15.20 ± 9.73	19.27	.000
11-13	13.46 ± 9.72		
14-17	12.93 ± 10.41		
Grade 1	15.00 ± /	7.87	.000
Grade 3	15.45 ± 9.88		
Grade 4	14.73 ± 9.61		
Grade 5	15.46 ± 9.58		
Grade 6	13.40 ± 9.55		
Grade 7	13.34 ± 9.88		
Grade 8	12.55 ± 10.04		
Grade 9	10.33 ± 17.04		
Grade 10	17.00 ± 14.14		

The participants assigned female sex at birth had significantly higher intrusion, avoidance, and total CRIES-8 scores. The difference was statistically significant using the Welch *t* test: *t*(4724.6) = −6.03, *p* < .001. The Wilcoxon test confirmed this result (*p* < 0.001). The highest CRIES-8 scores were observed in the youngest group (age 7-10 years), whereas the lowest scores were observed among adolescents (age 14-17 years). These findings suggest that in the war context, younger children may experience and report PTSD-related symptoms more intensely than older peers. ANOVA revealed significant differences in the academic performance of the youth (*F*_8,4744_ = 7.87, *p* < .001), confirmed by Kruskal–Wallis test (*p* < .001). Children in the lower grades (3-5) showed the highest PTSD symptoms.

Additional sociodemographic variables are indicated in Table 4. The item “Having a mother in the army” was omitted from this analysis because of having only one participant reply “yes” to this item. The item “Parent-reported ethnicity” was also omitted from this analysis, as only about 0.3% of the sample reported ethnicity other than Ukrainian. In regard to the number of children in the household, the Levene test revealed unequal variances across groups (*p* = .016). We found no significant differences between groups (standard ANOVA: *F*_8, 1503_ = 1.06, *p* = .388; Welch ANOVA: *p* =.088, Kruskal–Wallis *p* = .337).

**Table 4:** Child Revised Impact of Events Scale (CRIES-8) Scores and Their Differences in Sociodemographic Subgroups

	CRIES-8 intrusion			CRIES-8 avoidance			CRIES-8 total		
	Mean ± SD	*p*		Mean ± SD	*p*		Mean ± SD	*p*	
Having a single parent									
No	6.30 ± 5.28	.891		7.88 ± 6.00	.880		14.18 ± 9.98	.868	
Yes	6.37 ± 5.67			7.96 ± 5.70			14.33 ± 9.80		
Having a father in the army									
No	6.25 ± 5.25	.178		7.81 ± 5.94	.069		14.06 ± 9.90	.069	
Yes	6.92 ± 5.88			8.77 ± 6.24			15.70 ± 10.58		
Parent having a permanent job									
No	5.74 ± 5.30	.882		7.48 ± 6.09	.820		13.23 ± 10.22	.952	
Yes	5.79 ± 4.99			7.40 ± 5.83			13.19 ± 9.52		

	CRIES-8 intrusion			CRIES-8 avoidance			CRIES-8 total		
	Mean ± SD	*F*	*p*	Mean ± SD	*F*	*p*	Mean ± SD	*F*	*p*
Academic performance									
Low	6.02 ± 5.32	.780	.458	7.20 ± 5.60	.967	.380	13.22 ± 9.57	1.057	.348
Intermediate	6.44 ± 5.37			8.02 ± 5.99			14.46 ± 9.97		
High	6.14 ± 5.21			7.77 ± 5.99			13.91 ± 9.99		
Parental education									
Primary	7.40 ± 7.99	.274	.760	5.60 ± 5.18	.788	.455	13.00 ± 12.53	.146	.864
Secondary	5.73 ± 4.92			7.71 ± 5.92			13.44 ± 9.63		
Tertiary	5.79 ± 5.11			7.34 ± 5.89			13.12 ± 9.73		
Religion									
Non-religious	7.00 ± 5.82	.744	.475	9.16 ± 5.92	1.112	.329	16.16 ± 9.79	1.191	.304
Christian	5.76 ± 5.06			7.40 ± 5.89			13.16 ± 9.69		
Another religion	5.60 ± 5.06			7.10 ± 7.16			12.70 ± 12.01		
Parental marital status									
Not married	5.79 ± 4.83	1.683	.151	8.01 ± 5.82	1.493	.202	13.80 ± 9.42	1.788	.129
Married	5.71 ± 5.11			7.29 ± 5.93			13.00 ± 9.75		
Civil marriage	7.33 ± 4.73			9.33 ± 5.51			16.67 ± 10.21		
Divorced	7.78 ± 5.02			8.98 ± 4.95			16.75 ± 8.87		
Widowed	5.81 ± 3.83			8.75 ± 6.22			14.56 ± 9.24		
Language spoken in household									
Ukrainian	5.71 ± 5.05	4.173	.016	7.37 ± 5.91	2.328	.098	13.07 ± 9.70	3.980	.019
Russian	9.75 ± 4.65			10.75 ± 3.20			20.50 ± 7.14		
Both	7.49 ± 5.42			8.94 ± 5.45			16.43 ± 9.28		

The Kruskal–Wallis test indicated significant group differences in regard to the language spoken in the household, for intrusion (*p* = .016) and total (*p* = .017) scores, but not for avoidance (*p* = .060). Youth who came from Russian-speaking households had the highest scores on each subscale as well as the total score, followed by youth coming from bilingual households. However, interpretation should be made with caution, as one group (Russian, n = 4) was very small relative to the others, potentially inflating the observed differences.

The correlation of CRIES-8 intrusion, avoidance, and total scores and participants’ age in overall sample and the male and female subsample are indicated in Table 5. As shown in Table 5, younger age was weakly and yet significantly associated with higher intrusion, avoidance, and total CRIES-8 scores in both the overall sample and the subsample of participants assigned male sex at birth. In the female subsample, younger age also correlated significantly with higher avoidance and total scores, but intrusion did not show a significant association with age.

**Table 5:** Correlations of Age of Overall Sample and Male and Female Subsamples With Child Revised Impact of Events Scale (CRIES-8) Scores

	CRIES-8 intrusion		CRIES-8 avoidance		CRIES-8 total	
	r	p	r	p	r	p
Overall sample	–0.062	.000	–0.103	.000	–0.095	.000
Male	–0.097	.000	–0.117	.000	–0.123	.000
Female	–0.033	.098	–0.092	.000	–0.073	.000

Note: r = Pearson coefficient.

## Discussion

Our study aimed to estimate the prevalence of significant PTSD symptoms in children and adolescents aged 7 to 17 years in the city of Ternopil in Ukraine, and to examine the associations with putative demographic risk factors. Despite a lack of direct exposure to war-related traumas, we found that the prevalence of significant PTSD symptoms, based on recommended CRIES-8 cutoffs, was 40.3% in school children in Ternopil. Our data are in accordance with previously published data, indicating significant psychological trauma burden in children and adolescents living in war-affected regions.^9^ In a previous study, clinically significant PTSD symptoms were estimated to be present among 35% of adolescents aged 15 years and older in Ukraine.^9^ Our results indicate a higher prevalence of children screening positive for PTSD compared to the study by Osokina *et al.*^5^ conducted in 2 cities: Donetsk (5.3%), a region affected by war, and Kirovohrad (1.2%), a city in central Ukraine not affected by the war during that phase. These differences may partly reflect variations in measurement instruments and the mean sample age (13 years in their study vs 11 years in ours, with younger children showing higher PTSD symptoms). However, the higher prevalence observed in our sample may also reflect greater indirect exposure to the ongoing conflict among adolescents in present-day Ternopil. It is important to note that only a very small proportion of participants were internally displaced from more affected regions, making the observed prevalence particularly striking. At the same time, this assessment should be interpreted with caution, because CRIES-8 is a screening instrument, not a diagnostic tool, and the commonly used cutoff value of ≥17 may lead to an overestimation of clinically significant symptoms in large population-based screenings. In addition, youth may interpret questionnaire items differently depending on the context, and school administration may influence response patterns.

In our sample, positive screening for PTSD was more prevalent among female individuals (36.0% among boys and 44.2% among girls), which is consistent with data found in the literature, indicating that both psychosocial (eg, prior exposure to high-impact trauma, different coping strategies, and reliance on social support) and biological (eg, increased hypothalamic–pituitary–adrenal [HPA] axis reactivity, differences in neural circuitry, oxytocin-related mechanisms) factors contribute to this phenomenon.^15^ However, longitudinal research at ages 8, 10, 13, and 15 years showed a difference only in the oldest age group,^16^ suggesting the need to explore this disparity further and the reasons that may lead to it.

Our data indicate that younger age of the child significantly correlated with higher CRIES-8 scores. Although the literature suggests only a weak correlation between age and severity of PTSD, increasing the odds of adolescents experiencing a higher trauma burden compared to young adults,^17^ our findings raise the possibility that younger children may be more vulnerable to PTSD symptoms. One hypothesis is that adolescents have more mature cognitive appraisals and coping strategies,^18^ whereas younger children experience greater dependency, a reduced sense of control,^19^ and increased separation anxiety,^20^ all of which may elevate CRIES-8 scores. Young children’s reactions to trauma may also be mediated by threats to secure attachment relationships with caregivers, as their sense of security and emotional regulation are strongly dependent on the availability and stability of caregivers. In war contexts, heightened parental anxiety and perceived risk of separation from loved ones may further shape children’s psychological responses, especially in situations of prolonged social disruption such as the major invasion of Ukraine in 2022.^21^^,^^22^ However, it is also possible that part of the observed age difference reflects measurement-related factors. Younger children may use response scales differently from older participants, including a greater tendency toward extreme response options, which could contribute to higher reported scores in group-level comparisons.

Our findings also showed a link between lower academic achievement and higher levels of PTSD symptoms. This link may be bidirectional. Posttraumatic stress symptoms can impair concentration, memory, and engagement in school, which have negative impacts on academic achievement.^23^ Conversely, children with existing learning difficulties may experience greater stress and vulnerability in the context of ongoing conflict.^24^

Notably, this age effect differs from findings in other settings in which CRIES-8 has been administered outside of war contexts. For example, Salari *et al.*,^18^ in a study of unaccompanied refugee children in Sweden, and Duffy *et al.*,^19^ in a study of abused children in front-line social care services in the United Kingdom, did not report a significant association between age and CRIES-8 scores. These differences suggest that the unique context of war-related trauma may intensify the impact of age at exposure, with younger children showing particularly heightened vulnerability compared to peers in other adverse, but non–war-related, settings.

Interestingly, previous research indicated that posttraumatic symptoms in youth can show considerable variability in presentation, intensity, and course of the disorder.^20^ Our results indicate a higher burden of intrusions in girls compared to boys. Intrusion was also shown to be more prevalent in adult women compared to men with PTSD.^25^ This difference is postulated to be due to greater proneness to negative memory consolidation and recollection in female compared to male individuals and to greater thought suppression, which could be tied to the occurrence of intrusions.^25^ In addition, in conflict settings, girls may experience heightened perceived vulnerability to gender-based or sexual violence, which has been associated with increased threat anticipation and emotional memory encoding, potentially contributing to more frequent intrusive recollections.^26^^,^^27^

Although the evidence indicates that exposure to circumstances such as armed conflict or war-related events increases the risk of developing PTSD in children,^28^ it is important to mention several difficulties in comparing and interpreting data. The accurate estimate of the prevalence of PTSD in children in different regions is hindered by relatively low coverage of children’s and adolescents’ mental disorders in general, especially in low- to middle-income countries (LMICs).^29^ Furthermore, the choice of the diagnostic framework can be important in data interpretation.^30^ In addition, posttraumatic symptoms in youth can show considerable variability in presentation, intensity, and course of the disorder.^20^ Finally, in the previously reported studies, there is large variability in methodological approaches, such as sampling strategy, sample size, age range, and evaluation methods (structured clinical interviews or self-report questionnaires),^31^ which can influence data comparison. Notably, in assessing the prevalence of PTSD symptoms in war-related settings, the study performed by Osokina *et al.*^5^ relied on the Harvard Trauma Questionnaire (HTQ) in screening for PTSD, whereas that of Goto *et al.*^9^ used the Child and Adolescent Trauma Screen. Salari *et al.*,^18^ similar to our study, used CRIES-8, also relying on a cut-off value of 17 to determine clinical significance of PTSD symptoms. As previously mentioned, we have chosen CRIES-8 for this study because it is a brief measure, quick to complete, user-friendly for children, and designed as a screening tool to identify children at risk for PTSD.^11^

Therefore, our results should be interpreted cautiously because of several limitations. The design of our study is cross-sectional, thereby not allowing us to capture the fluctuations of posttraumatic symptoms in this population. In addition, the study design did not involve specifically measuring the exposure to war-related events or instructing children to base their self-reports on their war-related experiences; therefore, any PTSD symptoms cannot unequivocally be understood as war-related trauma symptoms. Finally, although the CRIES-8 instrument provides reliable screening for clinically significant PTSD symptoms, the design of this study did not involve further assessment through other instruments, such as structured clinical interviews, to establish related functional impairment and the diagnosis of this disorder.

The study shows a high prevalence of significant posttraumatic stress symptoms among children living in areas far from conflict zones. Therefore, our data suggest that the effects of war in regions not directly exposed may have a significant impact on youth mental health, suggesting an urgent need for the development and implementation of methods aimed at protecting and improving the mental health of children and adolescents in war-affected countries.

## CRediT authorship contribution statement

**Sanja Lestarevic Stojkov:** Writing – original draft, Data curation. **Marija Lero:** Writing – original draft, Data curation. **Svitlana Drin:** Formal analysis, Data curation. **Rachel Calam:** Supervision, Methodology, Conceptualization. **Andrea Danese:** Writing – review & editing. **Aala El-Khani:** Supervision, Methodology, Conceptualization. **Martha Fetsyk:** Validation, Data curation. **Halyna Nesmashna:** Supervision, Methodology, Conceptualization. **Halyna Radchuk:** Supervision, Methodology, Conceptualization. **Anna Tarasenko:** Supervision, Methodology, Conceptualization. **Kateryna Yavna:** Supervision, Methodology, Conceptualization. **John R. Weisz:** Writing – review & editing. **Dennis Ougrin:** Supervision, Methodology, Conceptualization.

## References

[1] Lewis S.J., Arseneault L., Caspi A. (2019). The epidemiology of trauma and post-traumatic stress disorder in a representative cohort of young people in England and Wales. Lancet Psychiatry.

[2] Danese A., McLaughlin K.A., Samara M., Stover C.S. (2020). Psychopathology in children exposed to trauma: detection and intervention needed to reduce downstream burden. BMJ.

[3] American Psychiatric Association (2013).

[4] Wallerich L., Fillol A., Rivadeneyra A., Vandentorren S., Wittwer J., Cambon L. (2023). Environment and child well-being: a scoping review of reviews to guide policies. Health Promot Perspect.

[5] Osokina O., Silwal S., Bohdanova T., Hodes M., Sourander A., Skokauskas N. (2023). Impact of the Russian invasion on mental health of adolescents in Ukraine. J Am Acad Child Adolesc Psychiatry.

[6] Scharbert J., Humberg S., Kroencke L. (2024). Psychological well-being in Europe after the outbreak of war in Ukraine. Nat Commun.

[7] UNICEF War in Ukraine: support for children and families. https://www.unicef.org/emergencies/war-ukraine-pose-immediate-threat-children.

[8] McElroy E., Hyland P., Shevlin M. (2023). Change in child mental health during the Ukraine war: evidence from a large sample of parents. Eur Child Adolesc Psychiatry.

[9] Goto R., Pinchuk I., Kolodezhny O., Pimenova N., Kano Y., Skokauskas N. (2024). Mental health of adolescents exposed to the war in Ukraine. JAMA Pediatr.

[10] Perrin S., Meiser-Stedman R., Smith P. (2005). The Children’s Revised Impact of Event Scale (CRIES): validity as a screening instrument for PTSD. Behav Cogn Psychother.

[11] Krause K.R., Chung S., Adewuya A.O. (2021). International consensus on a standard set of outcome measures for child and youth anxiety, depression, obsessive-compulsive disorder, and post-traumatic stress disorder. Lancet Psychiatry.

[12] Children and War Foundation (2025). Measures. https://www.childrenandwar.org/measures?utm_source=chatgpt.com.

[13] Deeba F., Rapee R.M., Prvan T. (2014). Psychometric properties of the Children’s Revised Impact of Events Scale (CRIES) with Bangladeshi children and adolescents. Peer J.

[14] Verlinden E., van Meijel E.P.M., Opmeer B.C. (2014). Characteristics of the Children’s Revised Impact of Event Scale in a clinically referred Dutch sample. J Trauma Stress.

[15] Olff M. (2017). Sex and gender differences in post-traumatic stress disorder: an update. Eur J Psychotraumatol.

[16] Haag K., Fraser A., Hiller R., Seedat S., Zimmerman A., Halligan S.L. (2020). The emergence of sex differences in PTSD symptoms across development: evidence from the ALSPAC cohort. Psychol Med.

[17] Karsberg S., Elklit A., Pedersen M.M., Pedersen M.U., Vang M.L. (2024). A nationally representative survey of ICD-11 PTSD among Danish adolescents and young adults aged 15–29. Scand J Psychol.

[18] Salari R., Malekian C., Linck L., Kristiansson R., Sarkadi A. (2017). Screening for PTSD symptoms in unaccompanied refugee minors: a test of the CRIES-8 questionnaire in routine care. Scand J Public Health.

[19] Duffy M., Walsh C., Mulholland C. (2021). Screening children with a history of maltreatment for PTSD in frontline social care organisations: an explorative study. Child Abuse Rev.

[20] Herd T., Haag A.C., Selin C. (2023). Individual and social risk and protective factors as predictors of trajectories of post-traumatic stress symptoms in adolescents. Res Child Adolesc Psychopathol.

[21] Bowlby J. (1982).

[22] Cobham V.E., McDermott B., Haslam D., Sanders M.R. (2016). The role of parents, parenting and the family environment in children’s post-disaster mental health. Curr Psychiatry Rep.

[23] Perfect M.M., Turley M.R., Carlson J.S. (2016). School-related outcomes of traumatic event exposure and traumatic stress symptoms in students: a systematic review of research from 1990 to 2015. Sch Ment Health.

[24] Bakhshi P., Babulal G.M., Trani J.F. (2018). Education and disability in a conflict-affected context: are children with disabilities less likely to learn and be protected in Darfur?. World Dev.

[25] Hsu C.M.K., Kleim B., Nicholson E.L. (2018). Sex differences in intrusive memories following trauma. PLoS One.

[26] Dellie E., Salelew E., Mihret Fetene S. (2024). Gender-based violence among women and girls in conflict-affected areas of Northeast Amhara, Ethiopia. Front Glob Womens Health.

[27] Maddox S.A., Hartmann J., Ross R.A., Ressler K.J. (2019). Deconstructing the gestalt: mechanisms of fear, threat, and trauma memory encoding. Neuron.

[28] Barenbaum J., Ruchkin V., Schwab-Stone M. (2004). The psychosocial aspects of children exposed to war: practice and policy initiatives. J Child Psychol Psychiatry.

[29] Erskine H.E., Baxter A.J., Patton G. (2017). The global coverage of prevalence data for mental disorders in children and adolescents. Epidemiol Psychiatr Sci.

[30] Dodd C.G., Kirk C.L., Rathouz P.J. (2024). Comparing diagnostic criteria for posttraumatic stress disorder in a diverse sample of trauma-exposed youth. J Trauma Stress.

[31] Carpiniello B. (2023). The mental health costs of armed conflicts—a review of systematic reviews conducted on refugees, asylum-seekers and people living in war zones. Int J Environ Res Public Health.

