# Supplementary material for: Prevalence and Correlates of Significant Posttraumatic Stress Disorder Symptoms in Children in Ukraine
Source: JAACAP Open. 2026 May 1;4(5):833–41. doi: 10.1016/j.jaacop.2026.04.007 (PMC13643461; doi:10.1016/j.jaacop.2026.04.007)
Supplement: Supplementary file 1 [file mmc1.docx]

Supplementary Table 1. Item-Total Correlations of CRIES-8

The internal consistency of the CRIES-8 was found to be within good range in this sample (Cronbach’s α = .825).

| Item | Corrected Item-Total Correlation | Cronbach’s Alpha If Item Deleted |
| --- | --- | --- |
| Do you think about it even when you don’t mean to? | .579 | .802 |
| Do you try to remove it from your memory? | .573 | .802 |
| Do you have waves of strong feelings about it? | .490 | .813 |
| Do you stay away from reminders of it (e.g. places or situations)? | .509 | .810 |
| Do you try not to talk about this event? | .539 | .807 |
| Do pictures about it pop into your mind? | .558 | .804 |
| Do other things keep making you think about it? | .529 | .808 |
| Do you try not to think about it? | .607 | .797 |

Item-total correlations were found to be within a very good range for all the items of CRIES-8.

*Supplementary Table 2. CRIES-8 intrusion and avoidance subscores and their differences in different sociodemographic subgroups – sex, age and grade (x̅±SD, (F), p values)*

|  | **CRIES-8 intrusion** | | | **CRIES-8 avoidance** | | |
| --- | --- | --- | --- | --- | --- | --- |
|  | **x̅±SD** | ***p*** | | **x̅±SD** | ***P*** | |
| **Sex assigned at birth** | | | | | | |
| Male | 5.83±5.05 | *.000* | | 7.36±5.94 | *.000* | |
| Female | 6.73±5.35 |  |  | 8.18±5.83 |  |  |
|  | | | | | | |
|  | **CRIES-8 intrusion** | | | **CRIES-8 avoidance** | | |
|  | **x̅±SD** | **F** | ***p*** | **x̅±SD** | **F** | ***p*** |
| **Age group** | | | | | | |
| 7 – 10 years old | 6.68±5.13 | 7.99 | .000 | 8.51±5.92 | 23.32 | *.000* |
| 11 – 13 years old | 6.06±5.24 |  |  | 7.40±5.82 |  |  |
| 14 – 17 years old | 6.05±5.53 |  |  | 6.88±5.99 |  |  |
| **Grade** | | | | | | |
| Grade 1 | 6.00±/ | 4.65 | *.000* | 9.00±/ | 8.53 | *.000* |
| Grade 3 | 6.88±5.21 |  |  | 8.58±5.98 |  |  |
| Grade 4 | 6.32±5.05 |  |  | 8.42±5.89 |  |  |
| Grade 5 | 6.97±5.14 |  |  | 8.49±5.78 |  |  |
| Grade 6 | 5.91±5.10 |  |  | 7.50±5.78 |  |  |
| Grade 7 | 5.95±5.26 |  |  | 7.39±5.95 |  |  |
| Grade 8 | 5.90±5.49 |  |  | 6.65±5.82 |  |  |
| Grade 9 | 5.67±8.96 |  |  | 4.67±8.08 |  |  |
| Grade 10 | 7.60±6.88 |  |  | 9.40±7.86 |  |  |
